# Impact of physical activity on disability‐free and disabled life expectancies in middle‐aged and older adults: Data from the healthy aging longitudinal study in Taiwan

**DOI:** 10.1111/ggi.14796

**Published:** 2024-01-02

**Authors:** Shu‐Chun Chuang, Yu‐Hung Chang, I‐Chien Wu, Yao‐Hwei Fang, Huei‐Ting Chan, Ray‐Chin Wu, Marion M. Lee, Chi‐Tsun Chiu, Hsing‐Yi Chang, Chao Agnes Hsiung, Chih‐Cheng Hsu

**Affiliations:** ^1^ Institute of Population Health Sciences National Health Research Institutes Miaoli Taiwan; ^2^ Department of Epidemiology and Biostatistics University of California San Francisco San Francisco California USA; ^3^ Institute of European and American Studies, Academia Sinica Taipei Taiwan; ^4^ Center for Geriatrics and Welfare Research National Health Research Institutes Yunlin Taiwan

**Keywords:** disability, life expectancy, physical activity at work

## Abstract

**Aim:**

Leisure‐time physical activity (LTPA) promotes healthy aging; however, data on work‐related physical activity (WPA) are inconsistent. This study was conducted to examine the disability‐free life expectancy (DFLE) and disabled life expectancy (DLE) across physical activity levels, with a focus on WPA, in middle‐aged and older adults.

**Methods:**

Data from 5663 community‐dwelling participants aged ≥55 years and enrolled in the Healthy Aging Longitudinal Study in Taiwan were evaluated. Energy expenditures from LTPA and WPA were calculated from baseline questionnaires and categorized into sex‐specific cutoffs. Disability was based on repeat measures of participants' activities of daily living and instrumental activities of daily living. Mortality was confirmed via data linkage with the Death Certificate database. DFLE and DLE were estimated from discrete‐time multistate life‐table models.

**Results:**

At age 65, women with low WPA had a DLE of 2.88 years (95% confidence interval [CI], 1.67–4.08), which was shorter than that of women without WPA (DLE, 5.24 years; 95% CI, 4.65–5.83) and with high WPA (DLE, 4.01 years; 95% CI, 2.69–5.34). DFLE and DLE were similar across WPA levels in men. DFLE tended to increase as the LTPA increased in men and women.

**Conclusion:**

Women with low WPA had shorter DLE than did those with no or high WPA. To reduce the risks of disability associated with physical activity, public policy should advocate for older people to watch the type, amount, and intensity of their activities as these may go ignored during WPA. **Geriatr Gerontol Int 2024; 24: 229–239**.

## Graphical abstract

We used data from the Healthy Aging Longitudinal Study in Taiwan to evaluate the association between type of activities (leisure‐time and work‐related) and disability‐free and disabled life years. Our study suggested that work‐related physical activity was U‐shaped associated with disabled life years in women. The results implied that older people should watch the type, amount, and intensity of their activities as these may go ignored during work.

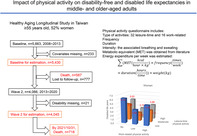

## Introduction

In Taiwan, life expectancy at birth increased from 62 years in 1960 to 78 years in 2020 for men and from 66 years in 1960 to 85 years in 2020 for women.^1^ However, living longer does not mean living healthily. Thus, disability‐free life expectancy (DFLE) and disabled life expectancy (DLE) were proposed as summary measures of a population's health, considering the quality and quantity of lived years.^2^

Physical activity can benefit the health of older populations by reducing total mortality, cardiovascular morbidity and mortality, diabetes incidence, and the risk of some cancers (e.g., breast and colorectal)^3^, ^4^; however, most previous studies focused only on leisure‐time physical activity (LTPA). Studies have indicated that occupational physical activity may be associated with a higher risk of cardiovascular disease and mortality; this phenomenon has been termed the *physical activity paradox*.^5^, ^6^, ^7^, ^8^, ^9^, ^10^, ^11^ Occupational physical activity is characterized by static and isometric activities, for example, standing and lifting for a long period of time, and without sufficient recovery time.^12^, ^13^ Hours of higher levels of occupational physical activity can lead to prolonged increases in heart rate and blood pressure, thus triggering adverse intravascular turbulence and increasing the risk for cardiovascular diseases and mortality. Additionally, the working environment might be out of the workers' control and thus may increase stress and inflammation levels^9^, ^14^ as well as injuries. Nevertheless, the physical activity paradox seems to be more apparent in men and those with poor cardiorespiratory fitness.^15^

Previous studies have suggested that physical activity contributes to a longer life expectancy, either independently or jointly with other healthy lifestyles.^16^, ^17^, ^18^, ^19^ However, few studies have investigated the impact of occupational physical activity on DFLE and DLE. Researchers in Finland examined the association between physical activity (including leisure‐time and commuting activities) and healthy and chronic disease‐free life expectancy by occupational status.^17^ They found that the gain in healthy and chronic disease‐free years with higher physical activity was larger among persons with lower than with higher occupational status.^17^ However, occupational status does not necessarily correspond to work‐related physical activity (WPA). Although seniors may have retired from their jobs, they may still engage in WPAs, such as farming, gardening, or babysitting, or mix WPA and LTPA. Health effects from these types of WPAs in older populations should be evaluated.

This study was conducted to examine the effect of WPA on DFLE and DLE by examining the association between LTPA and WPA, individually and combined, and DFLE and DLE by sex. As socioeconomic status and underlying morbidities often confound the association between work and health,^15^ the secondary aim of the study was to present the DFLE and DLE by WPA in subgroups with different educational attainment and selected morbidities.

## Methods

### The Healthy Aging Longitudinal Study in Taiwan

Data were derived from the Healthy Aging Longitudinal Study in Taiwan (HALST), a prospective study established in 2008, in which 5663 community‐dwelling older adults were recruited across Taiwan. The second wave of data collection began in 2013 and ended in 2020, with a response rate of 73.6%. The cohort has been described previously.^20^ Briefly, a random sample of eligible residents (≥55 years old) living within the catchment area of seven collaborative hospitals were recruited. Participants with any of the following conditions were excluded: highly contagious infectious diseases, diagnosed dementia, severe illness (based on the interviewers' judgment of whether the participant was too ill to complete the interview), being bedridden, severe mental disorders, mutism, hearing impairment, blindness, living in a long‐term care facility, or being hospitalized. Detailed illustration of participant selection is presented in Figure S1. All participants signed written informed consent. The Institutional Review Board at the National Health Research Institutes and the collaborative hospitals approved the study. All methods were performed in accordance with the relevant guidelines and regulations.

### Measurement of physical activity

We adapted the physical activity questionnaire used in the National Health Interview Survey in Taiwan.^21^ Information on LTPA and WPA were collected for all HALST participants. Each participant was asked whether they had engaged in LTPA or WPA during the past year. If yes, the participant was then asked to select up to five activities from a list of 32 LTPAs and 16 WPAs (Table S1). Frequency, duration, and associated breathing and sweating effects were recorded for each of the five activities selected. For each physical activity, a metabolic equivalent (MET) value was assigned according to the level of perceived breathing effects following the recommendations of Wai *et al*.^22^ and Ainsworth *et al*.^23^ Energy expenditure from physical activity, summarizing duration, frequency, and intensity for an individual on a weekly basis (kcal/week) was calculated as:

$$\sum \left(\mathrm{MET}\left(\frac{\text{kcal}}{\text{hour}\times \mathrm{kg}}\right)\times \text{frequency}\left(\frac{\text{times}}{\text{week}}\right)\times \text{duration}\left(\frac{\text{hours}}{\text{time}}\right)\right)\times \text{weight}\left(\mathrm{kg}\right)$$

where weight was measured during the physical examination by the trained interviewers.

The LTPA was then categorized according to sex‐specific tertile. Approximately 75% of our cohort had participated in no WPA during the previous year; thus, WPA was categorized as none, low, or high, according to the sex‐specific median. Energy expenditure from leisure‐time and work‐related physical activities were summed to obtain the total physical activity and categorized according to sex‐specific tertile.

### Outcome measures

#### Disability

In both waves of data collection, participants were asked whether they had difficulty performing activities of daily living (ADL)^24^ and instrumental activities of daily living (IADL).^25^ ADL assesses functional status, which includes eating, getting in/out of bed, personal hygiene, grooming, using the toilet, bathing/showering, walking across a room, ascending/descending stairs, dressing, controlling bowel movements, and controlling urination. ADL scores range from 0 to 100, with 100 representing full independence in doing daily activities.^24^ The IADL measures independent living skills, including shopping, housekeeping, handling finances, food preparation, transportation, telephone use, laundry, and responsibility for taking medication.^25^ In Taiwanese culture, men are not required to do housekeeping, food preparation, or laundry; thus, these three items were not scored for men. Thus, IADL scores ranged from 0 (worst) to 8 (best) for women and 0–5 for men. Disabled persons were defined as those whose scores were <100 on the ADL or <8 for women and <5 for men on the IADL.

#### Mortality

Participants' vital statuses and cause and date of death were confirmed via data linkage with the Taiwan National Death Certificate database updated on October 31, 2021.

### Measurements of other variables

Other covariates considered in this study were study centers, education levels (low literacy/did not attend primary school, attended or completed primary school, or more than primary school), smoking status, alcohol consumption status (never, former, or current), body mass index, social network score, Center for Epidemiologic Studies Depression Scale scores (<16 or ≥16), total energy intake (kcal), and selected health conditions.^7^, ^8^, ^9^, ^10^, ^15^, ^26^, ^27^, ^28^, ^29^, ^30^, ^31^, ^32^

Most of these variables and health conditions were self‐reported, except height, weight (for calculation of body mass index), and waist circumference, which were measured during the physical examination by the trained interviewers. The social network score was calculated on the basis of six questions about the participant's interactions with immediate family members, relatives, friends, and neighbors, and about participation in community activities. Each question was scored from 0 to 2, with a higher score indicating higher frequency of interaction or participation. A social network score is the summary score of the six questions (range, 0–12). The metabolic syndrome was defined as having any three of the following conditions^33^: (i) abdominal obesity, that is, waist circumference ≥90 cm in men or ≥ 80 cm in women; (ii) elevated triglycerides, that is, fasting serum triglycerides ≥150 mg/dL or self‐reported treatments for dyslipidemia; (iii) reduced high‐density lipoprotein cholesterol, that is, <40 mg/dL in men and < 50 mg/dL in women or self‐reported treatments for dyslipidemia; (iv) elevated blood pressure (BP), that is, systolic BP ≥130 mm Hg or diastolic BP ≥85 mm Hg or self‐reported treatments for hypertension; and (v) abnormal glucose metabolism, that is, fasting glucose ≥100 mg/dL or self‐reported treatment for diabetes. Household income was also available in the HALST. However, almost half of the participants (45.8%) reported unknown or refused to answer the question. Hence, household income was not included in the statistical models but only for subgroup analyses. Interpretation of the results by household income should be cautious.

### Statistical analysis

Only data on completed physical activities, disability, and covariates were considered in the analysis. A flowchart of data included in the current analyses is illustrated in Figure S2. Participants' characteristics were expressed as counts (%) and compared using chi‐square tests for categorical variables or described as means ± standard deviations and compared using the Kruskal–Wallis test for continuous variables.

We applied the Stochastic Population Analysis for Complex Events program,^34^ a multistate life table (MSLT) method to compute disability‐free years from the time of no disability to disability and death. Three health states were defined: disability‐free, disability, and death. Four possible transitions were allowed between the states: disability‐free to disability, disability to disability‐free, disability‐free to death, and disability to death. An age‐specific transition probability was estimated from the HALST study for all possible transitions using multinomial logistic regression with sex, physical activities, and all other covariates in the model. LTPA and WPA were mutually adjusted in all models. Total life expectancy (TLE), DFLE, and DLE were then calculated on the basis of these estimated transition probabilities using a stochastic (microsimulation) approach. A simulated cohort of 100 000 individuals was generated according to the distribution of covariates at baseline based on observed prevalence in the HALST study by age group, sex, and physical activity level. TLE, DFLE, and DLE were then summarized from these simulated results for men and women and for physical activity level. Standard errors and their corresponding 95% confidence intervals (CIs) were calculated using a bootstrap method with 100 repeats for the entire analysis process (multinomial analysis and stimulation steps). Significant differences between physical activity levels were tested using a two‐sample Z‐test.

All analyses were performed using SAS 9.4 (SAS Institute, Cary, NC, USA).

## Results

We used data from 5430 participants at baseline and 4045 participants at follow‐up. Table 1 presents the baseline characteristics by WPA level for both sexes. Both men and women with higher WPA levels were younger and tended to have lower education and LTPA levels. Disability prevalence was approximately 12% in men and 19% in women; the prevalence was lower in those with higher WPA levels. Baseline characteristics for participants by follow‐up status are presented in Table S2.

**Table 1:** Baseline characteristics of participants by work‐related physical activity

	Men							Women						
	No (*n* = 1925)		Low (*n* = 328)		High (*n* = 330)		*p*	No (*n* = 2133)		Low (*n* = 357)		High (*n* = 357)		*P* value
	*N*	%	*N*	%	*N*	%		*N*	%	*N*	%	*N*	%	
Age (years, mean ± SD)	70.61	8.61	68.10	7.47	67.37	7.58	<0.01	69.67	8.05	67.43	6.79	67.57	7.83	<0.01
BMI (kg/m^2^, mean ± SD)	24.49	3.33	24.39	3.24	24.69	3.23	0.31	24.62	3.67	24.16	3.41	24.68	3.85	0.08
Energy intake (kcal, mean ± SD)	2256.25	781.96	2363.64	777.76	2361.08	795.47	0.01	1787.92	631.63	1808.12	606.12	1863.36	651.48	0.16
Education levels														
Low literacy	55	2.9	8	2.4	13	3.9	<0.01	317	14.9	62	17.4	93	26.1	<0.01
Primary school	707	36.7	128	39.0	188	57.0		1031	48.3	168	47.1	195	54.6	
More than primary school	1163	60.4	192	58.5	129	39.1		785	36.8	127	35.6	69	19.3	
Household income														
<30 K NTD	606	31.5	103	31.4	128	38.8	0.01	628	29.4	127	35.6	138	38.7	<0.01
30 K–< 70 K NTD	290	15.1	56	17.1	44	13.3		227	10.6	38	10.6	41	11.5	
≥70 K NTD	238	12.4	54	16.5	30	9.1		157	7.4	29	8.1	9	2.5	
Unknown or refused to answer	791	41.1	115	35.1	128	38.8		1121	52.6	163	45.7	169	47.3	
Smoking														
Never	820	42.6	151	46.0	131	39.7	0.11	2079	97.5	351	98.3	355	99.4	0.13
Former	637	33.1	104	31.7	99	30.0		15	0.7	3	0.8	1	0.3	
Current	468	24.3	73	22.3	100	30.3		39	1.8	3	0.8	1	0.3	
Drinking														
Never	758	39.4	106	32.3	134	40.6	<0.01	1728	81.0	265	74.2	292	81.8	0.01
Former	393	20.4	50	15.2	37	11.2		71	3.3	12	3.4	6	1.7	
Current	774	40.2	172	52.4	159	48.2		334	15.7	80	22.4	59	16.5	
Physical activity at leisure time														
Low	531	27.6	116	35.4	198	60.0	<0.01	620	29.1	113	31.7	191	53.5	<0.01
Mediate	710	36.9	94	28.7	72	21.8		750	35.2	119	33.3	91	25.5	
High	684	35.5	118	36.0	60	18.2		763	35.8	125	35.0	75	21.0	
Social networking														
≥8	844	43.8	203	61.9	194	58.8	<0.01	994	46.6	196	54.9	205	57.4	<0.01
6–7	559	29.0	72	22.0	81	24.5		605	28.4	103	28.9	89	24.9	
0–5	522	27.1	53	16.2	55	16.7		534	25.0	58	16.2	63	17.6	
CESD														
<16	1840	95.6	324	98.8	320	97.0	0.01	1961	91.9	343	96.1	337	94.4	0.01
≥16	85	4.4	4	1.2	10	3.0		172	8.1	14	3.9	20	5.6	
Prevalence of chronic conditions														
Disability	262	13.6	20	6.1	21	6.4	<0.01	452	21.2	48	13.4	54	15.1	<0.01
Hypertensive medication	839	43.6	110	33.5	113	34.2	<0.01	921	43.2	142	39.8	122	34.2	<0.01
Dyslipidemia medication	276	14.3	24	7.3	24	7.3	<0.01	385	18.0	51	14.3	36	10.1	<0.01
Diabetic medication	374	19.4	36	11.0	43	13.0	<0.01	374	17.5	41	11.5	39	10.9	<0.01
Metabolic syndrome	862	45.7	114	35.0	114	35.0	<0.01	1170	55.9	174	48.9	181	51.1	0.02
Stroke	155	8.1	10	3.1	12	3.6	<0.01	98	4.6	6	1.7	5	1.4	<0.01
Cancer	113	5.9	8	2.4	9	2.7	<0.01	159	7.5	18	5.0	11	3.1	<0.01
Gout	344	17.9	45	13.7	40	12.1	0.01	141	6.6	20	5.6	11	3.1	0.03
Hip fracture	37	1.9	2	0.6	3	0.9	0.12	40	1.9	3	0.8	5	1.4	0.34
Other musculoskeletal diseases	625	32.5	93	28.4	89	27.0	0.07	1142	53.5	163	45.7	184	51.5	0.02

Abbreviations: CESD, Center for Epidemiologic Studies Depression Scale; SD, standard deviation.

By the end of the follow‐up, 1305 participants had died (799 men and 506 women). Table 2 shows the TLE, DFLE, and DLE for both men and women at the ages of 55, 65, and 75 years, by physical activity level. Overall, WPA was nonlinearly associated with DLE, particularly in women. At age 65, women with low WPA had a DLE of 2.88 years (95% CI, 1.67–4.08), which was shorter than that of women without WPA (DLE, 5.24 years; 95% CI, 4.65–5.83) and with high WPA (DLE, 4.01 years; 95% CI, 2.69–5.34).

**Table 2:** Total, disability‐free, and disabled life expectancies

		Total life expectancy		Disability‐free life expectancy		Disabled life expectancy		Net gain or loss					
								Total life expectancy		Disability‐free life expectancy		Disabled life expectancy	
Age		Years	95% CI	Years	95% CI	Years	95% CI	Years	95% CI	Years	95% CI	Years	95% CI
Men													
Physical activity at leisure time													
At 55	Low	24.39	(23.36 to 25.42)	22.24	(21.05 to 23.44)	2.15	(1.41 to 2.89)	Ref		Ref		Ref	
	Moderate	24.94	(23.74 to 26.14)	22.25	(20.95 to 23.55)	2.69	(1.90 to 3.47)	0.55	(−1.04 to 2.13)	0.01	(−1.76 to 1.78)	0.54	(−0.54 to 1.62)
	High	26.81	(25.65 to 27.96)	24.58	(23.42 to 25.73)	2.23	(1.53 to 2.93)	2.41	(0.86 to 3.96)	2.33	(0.67 to 3.99)	0.08	(−0.94 to 1.10)
At 65	Low	16.20	(15.43 to 16.96)	14.02	(13.10 to 14.94)	2.18	(1.59 to 2.77)	Ref		Ref		Ref	
	Moderate	16.63	(15.67 to 17.59)	14.14	(13.18 to 15.10)	2.49	(1.88 to 3.09)	0.43	(−0.79 to 1.66)	0.12	(−1.21 to 1.45)	0.31	(−0.53 to 1.15)
	High	18.17	(17.18 to 19.16)	16.08	(15.15 to 17.01)	2.09	(1.50 to 2.68)	1.97	(0.72 to 3.22)	2.06	(0.75 to 3.37)	−0.09	(−0.92 to 0.75)
At 75	Low	9.43	(8.89 to 9.98)	7.20	(6.57 to 7.83)	2.23	(1.81 to 2.65)	Ref		Ref		Ref	
	Moderate	9.77	(9.07 to 10.47)	7.51	(6.86 to 8.15)	2.27	(1.81 to 2.72)	0.34	(−0.55 to 1.23)	0.31	(−0.60 to 1.21)	0.03	(−0.58 to 0.65)
	High	10.92	(10.12 to 11.71)	8.98	(8.26 to 9.69)	1.94	(1.47 to 2.41)	1.48	(0.52 to 2.45)	1.77	(0.82 to 2.73)	−0.29	(−0.92 to 0.34)
Physical activity at work													
At 55	No	25.07	(24.25 to 25.89)	22.73	(21.78 to 23.68)	2.34	(1.80 to 2.88)	Ref		Ref		Ref	
	Low	26.34	(24.56 to 28.11)	24.10	(22.32 to 25.88)	2.24	(1.09 to 3.39)	1.26	(−0.69 to 3.22)	1.36	(−0.65 to 3.38)	−0.10	(−1.37 to 1.17)
	High	25.32	(23.68 to 26.96)	22.76	(20.93 to 24.59)	2.56	(1.42 to 3.70)	0.25	(−1.59 to 2.08)	0.03	(−2.03 to 2.09)	0.22	(−1.05 to 1.48)
At 65	No	16.75	(16.14 to 17.35)	14.51	(13.88 to 15.15)	2.23	(1.82 to 2.64)	Ref		Ref		Ref	
	Low	17.81	(16.29 to 19.34)	15.73	(14.26 to 17.20)	2.08	(1.11 to 3.06)	1.07	(−0.57 to 2.71)	1.22	(−0.38 to 2.82)	−0.15	(−1.21 to 0.91)
	High	16.92	(15.49 to 18.34)	14.63	(13.20 to 16.06)	2.28	(1.34 to 3.23)	0.17	(−1.38 to 1.72)	0.12	(−1.45 to 1.68)	0.05	(−0.98 to 1.08)
At 75	No	9.81	(9.40 to 10.23)	7.64	(7.21 to 8.06)	2.18	(1.86 to 2.49)	Ref		Ref		Ref	
	Low	10.77	(9.56 to 11.98)	8.70	(7.60 to 9.80)	2.08	(1.28 to 2.87)	0.96	(−0.32 to 2.24)	1.06	(−0.12 to 2.24)	−0.10	(−0.96 to 0.76)
	High	9.98	(8.82 to 11.14)	7.94	(6.95 to 8.93)	2.04	(1.27 to 2.80)	0.17	(−1.06 to 1.40)	0.31	(−0.77 to 1.38)	−0.14	(−0.96 to 0.68)
Women													
Physical activity at leisure time													
At 55	Low	29.54	(28.50 to 30.57)	23.09	(21.61 to 24.58)	6.45	(5.31 to 7.58)	Ref		Ref		Ref	
	Moderate	29.75	(28.71 to 30.80)	25.77	(24.64 to 26.91)	3.98	(2.92 to 5.04)	0.22	(−1.26 to 1.69)	2.68	(0.81 to 4.55)	−2.47	(−4.02 to −0.92)
	High	30.08	(28.90 to 31.25)	25.09	(23.91 to 26.28)	4.98	(3.87 to 6.09)	0.54	(−1.03 to 2.11)	2.00	(0.10 to 3.90)	−1.47	(−3.06 to 0.12)
At 65	Low	20.05	(19.13 to 20.97)	14.16	(13.05 to 15.27)	5.89	(5.05 to 6.73)	Ref		Ref		Ref	
	Moderate	20.16	(19.21 to 21.12)	16.30	(15.38 to 17.21)	3.87	(3.03 to 4.70)	0.11	(−1.21 to 1.44)	2.14	(0.70 to 3.58)	−2.02 to	(−3.21 to −0.84)
	High	20.52	(19.38 to 21.66)	15.98	(14.98 to 16.98)	4.54	(3.62 to 5.46)	0.47	(−1.00 to 1.94)	1.82	(0.32 to 3.32)	−1.35	(−2.60 to −0.10)
At 75	Low	11.63	(10.91 to 12.36)	6.97	(6.24 to 7.69)	4.67	(4.05 to 5.29)	Ref		Ref		Ref	
	Moderate	11.68	(10.87 to 12.49)	8.27	(7.61 to 8.93)	3.41	(2.82 to 4.00)	0.05	(−1.04 to 1.14)	1.30	(0.33 to 2.28)	−1.26	(−2.11 to −0.40)
	High	12.02	(11.00 to 13.03)	8.24	(7.46 to 9.03)	3.78	(3.02 to 4.53)	0.38	(−0.87 to 1.63)	1.27	(0.21 to 2.34)	−0.89	(−1.87 to 0.08)
Physical activity at work													
At 55	No	29.92	(29.10 to 30.73)	24.21	(23.15 to 25.26)	5.71	(4.91 to 6.51)	Ref		Ref		Ref	
	Low	28.35	(26.70 to 30.00)	25.33	(23.50 to 27.17)	3.02	(1.70 to 4.34)	−1.56	(−3.40 to 0.28)	1.13	(−0.99 to 3.25)	−2.69	(−4.23 to −1.15)
	High	30.73	(28.88 to 32.58)	26.59	(24.46 to 28.72)	4.14	(2.55 to 5.73)	0.81	(−1.21 to 2.83)	2.39	(0.01 to 4.76)	−1.57	(−3.35 to 0.20)
At 65	No	20.37	(19.64 to 21.10)	15.13	(14.35 to 15.91)	5.24	(4.65 to 5.83)	Ref		Ref		Ref	
	Low	18.94	(17.42 to 20.47)	16.07	(14.39 to 17.75)	2.88	(1.67 to 4.08)	−1.43	(−3.12 to 0.26)	0.94	(−0.91 to 2.79)	−2.37	(−3.70 to −1.03)
	High	21.22	(19.48 to 22.96)	17.20	(15.38 to 19.02)	4.01	(2.69 to 5.34)	0.85	(−1.04 to 2.73)	2.07	(0.09 to 4.06)	−1.23	(−2.68 to 0.22)
At 75	No	11.87	(11.28 to 12.47)	7.57	(7.04 to 8.10)	4.30	(3.82 to 4.79)	Ref		Ref		Ref	
	Low	10.71	(9.43 to 11.99)	8.03	(6.78 to 9.27)	2.68	(1.83 to 3.63)	−1.17	(−2.58 to 0.24)	0.46	(−0.90 to 1.81)	−1.62	(−2.69 to −0.56)
	High	12.61	(11.15 to 14.07)	9.02	(7.72 to 10.32)	3.59	(2.60 to 4.58)	0.74	(−0.84 to 2.31)	1.45	(0.05 to 2.85)	−0.71	(−1.82 to 0.39)

Abbreviation: CI, confidence interval.

**Figure 1:**
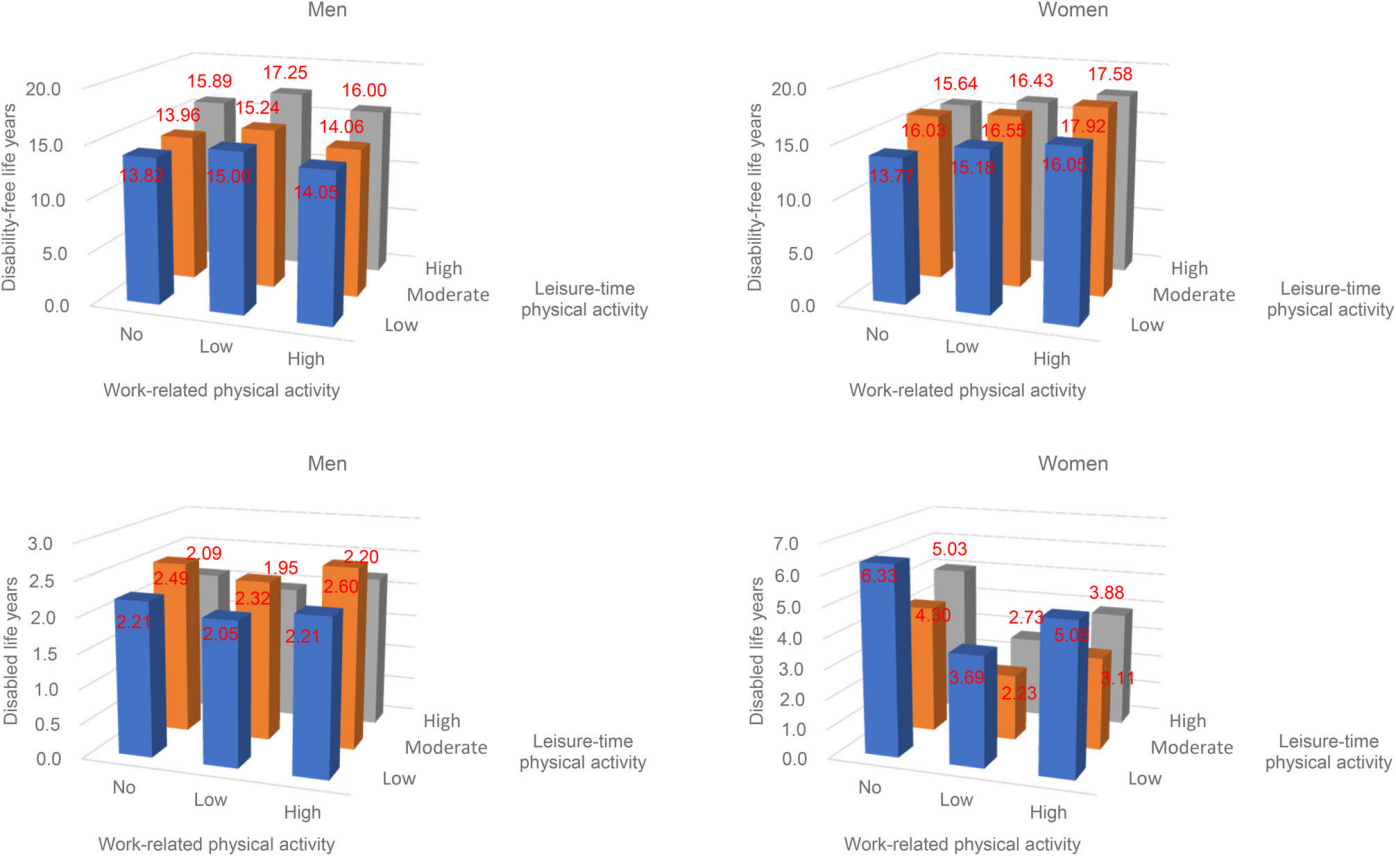
Disability‐free and disabled life expectancies at 65 years old by leisure‐time and work‐related physical activity levels in men and women.

Figure 1 displays the combined effect of LTPA and WPA on DFLE and DLE at age 65. Overall, LTPA and WPA showed a U‐shaped association with DLE in women. Women with moderate LTPA and a lower WPA had the shortest DLE (2.23 years; 95% CI, 1.22–3.23).

**Figure 2:**
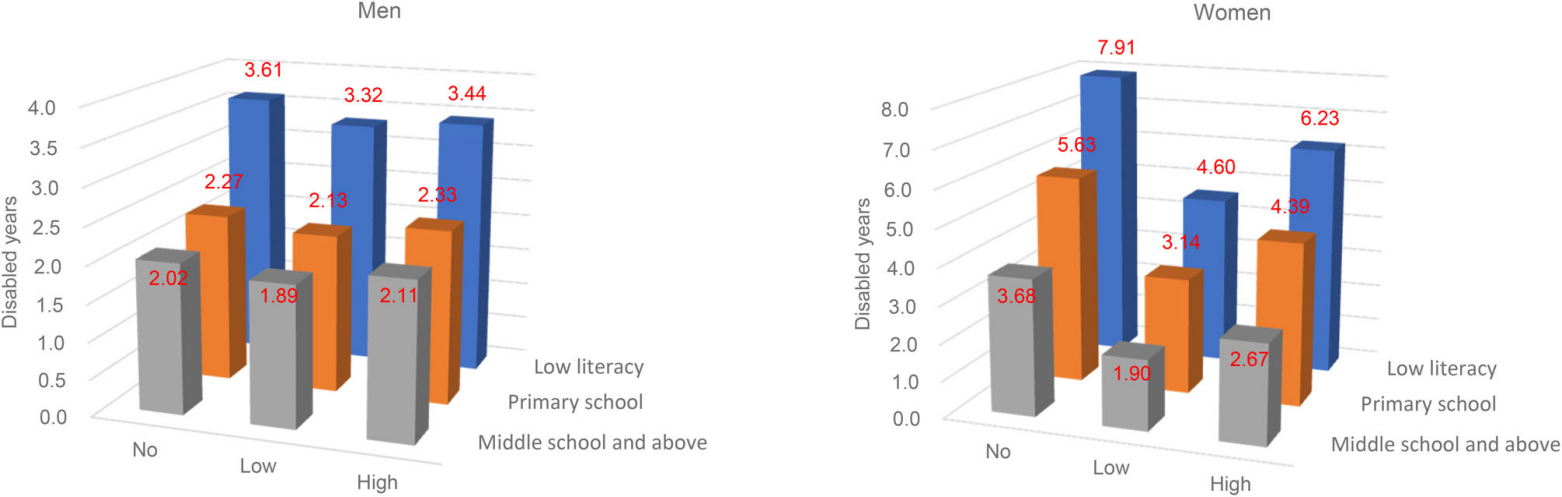
Disabled life expectancies at 65 years old by education and work‐related physical activity levels in men and women.

**Figure 3:**
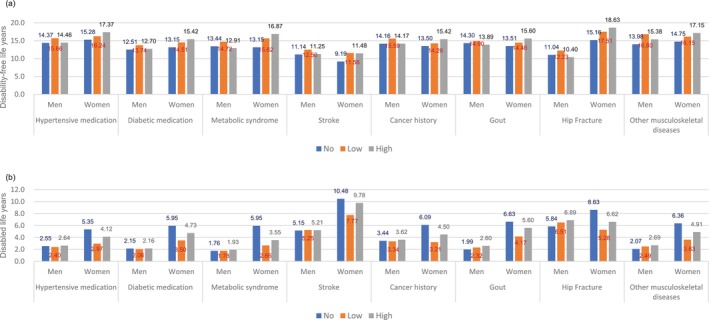
Disability‐free (a) and disabled (b) life expectancies at 65 years by work‐related physical activity level and baseline health conditions. Other musculoskeletal diseases included osteoporosis, arthritis, and spurs.

We also conducted subgroup analyses by education level and baseline health conditions. The length of time living with a disability increased as the education level decreased in both men and women (Figure 2). However, WPA remained nonlinearly associated with DLE in women regardless of education level. In participants with previous health conditions, the U‐shaped association between WPA and DLE remained in women with hypertension, diabetes, and metabolic syndrome (Figure 3).

## Discussion

In the current study, WPA and DLE were nonlinearly associated in women, and women with low WPA and moderate LTPA had the shortest DLE. In 2014, the midpoint of our study, the published life expectancies at age 65 were 18 years for men and 21 years for women.^1^ The estimated TLE from the current longitudinal cohort study was 17 years for men and 20 years for women.

In addition to morbidity and mortality, physical activity has been associated with a healthy aging trajectory,^35^ reduced cognitive decline,^36^ and reduced frailty progression.^37^ Again, these studies were based on LTPA. Few studies have investigated the impact of WPA on functional changes in older people. The Mayo Clinic Study of Aging suggested that moderate‐ or heavy‐intensity nonexercise physical activity was associated with less cognitive loss.^38^ However, that study did not adjust for LTPA.^38^ Conversely, the Copenhagen Male Study found that men with high WPA levels had a statistically significant increased risk of dementia compared to that of men with sedentary jobs.^39^ Previous studies on LTPA indicated that physical activity may benefit certain cognitive domains, such as executive function and memory, but not attention or working memory.^40^ Different types of WPA may also affect different domains of cognition. Overall, decreased cognition and advanced frailty may result in disability and poor quality of life. More studies on WPA and functions are needed.

While previous studies on WPA and all‐cause mortality or cardiovascular disease risk were more pronounced in men than in women,^15^, ^41^ we did not observe an association between WPA and DFLE and DLE in men. The explanations for sex differences in mortality included differences in physically demanding jobs performed by men and women, different exposures resulting from these different jobs, different perceived intensity of WPA by men and women, different gender responses to cardiovascular risk factors and WPA, and interactions between tasks undertaken at the workplace and at home.^42^, ^43^, ^44^, ^45^, ^46^ In other words, if men with physically demanding jobs had a higher chance of premature death, we may have less probability to recruit such men to our study. Men involved in the current study may have more survival advantages than the general population. As shown in Table S4, data from the National Health Interview Survey, a representative sample of Taiwan's general population, showed that the energy expenditure from WPA was the highest in 35‐ to 54‐year‐old men and 55‐ to 74‐year‐old women. The energy expenditure from WPA reported in the current study might not reflect lifelong energy expenditures from WPA, particularly in men. This may explain why we observed no association between WPA and DFLE or DLE in men.

On the other hand, the energy expenditure from WPA reported in the current study might capture the lifetime peak in women (Table S4). Women without WPA may already present disability or chronic diseases (Table 1) that prevent them from working and result in longer DLE. Healthy women were able to participate in WPA; hence, related to decreased DLE. However, our results also indicated that DLE increased when WPA increased, although the increase was not statistically significant. Indeed, although WPA was not related to mortality in women, a systemic review suggested that men and women have different risks of work‐related injuries and disabilities.^47^ Some studies found that women had a higher risk of musculoskeletal disorders and injuries than men in similar occupations.^48^, ^49^, ^50^ The design of working environment that was usually for anthropometric average men^51^ and the double burden from both work and family responsibility^52^ may explain the gender differences in disability and chronic conditions. Furthermore, the nonassociation between occupation and mortality in women might be because the previous occupational health studies were usually underrepresented by women, particularly in occupations that were dominated by men.^47^ As the DLE measurement combines disability and mortality, the nonlinear association between WPA and DLE in women may highlight the impact of work‐related disability in women.

For easier interpretation of the nonlinear association, we translated the energy expenditure into minutes per week for a 70‐kg man and a 60‐kg woman. The lower and higher LTPA cutoffs for men were 41 and 299 min/week, respectively, for moderate exercise (e.g., walking MET 3.5); or 24 and 174 min/week, respectively, for vigorous exercise (e.g., jogging MET 6.0). For women, those cutoffs were 37 and 240 min/week, respectively, for moderate exercise and 21 and 140 min/week, respectively, for vigorous exercise. The WPA cutoffs were 310 and 311 min/week for regular WPA (e.g., farming, heavy housekeeping work, or babysitting [MET 4.0]) for men and women, respectively. Energy expenditure in high levels of WPA were usually from farming and manual work in men and farming and housekeeping in women. The World Health Organization recommends that older adults perform an equivalent combination of moderate and vigorous activities ≥150 min, or even >300 min for additional health benefits.^53^ Our findings were consistent with the World Health Organization's recommendation that higher LTPA levels were associated with longer DFLE. However, our results also indicated that higher WPA levels may provide no additional health benefit, particularly in women.

After combining LTPA and WPA as total physical activity, the nonlinear association between total physical activity and DLE remained in women, and the association between total physical activity and higher DFLE was not statistically significant (Table S3). Higher total physical activity was resulted from the higher proportion of WPA in both men and women (data not shown). As per Physical Activity Guidelines for Americans,^54^ injuries and other adverse events, such as overheating, dehydration, musculoskeletal injuries, or stroke, may occur during activities and may increase the risk of disability, thus increasing the DLE.

In addition, those who worked for longer hours might be those who need more income. Previous systemic reviews on employment in later life suggested that working helps to promote and maintain health in older adults.^55^, ^56^ However, motivation, financial status, and physical health were important effect modifiers.^55^, ^57^, ^58^, ^59^ Research from the Longitudinal Aging Study Amsterdam suggested different life expectancy by different occupation and occupational exposures.^60^, ^61^ These studies revealed a need to provide specific support regarding the working environment, and the pension policy should be tailored according to individuals' risk of longevity. However, the WPA in our study does not correspond to occupation or occupational exposure in the previous studies. Nevertheless, our subgroup analyses showed that the U‐shaped association between WPA and DLE remained across women with different household income (Figure S3).

A previous study from the Taiwan Longitudinal Study on Aging suggested that higher education, exercise, employment, and social participation were associated with lower risks of early onset of disability.^62^ Studies from Europe and South America suggest that education affects choices in occupation and health behaviors, including physical activity.^63^, ^64^ The results from the Health and Retirement Study further demonstrated that higher education was associated with compression of mortality and disability.^65^ A multicohort study in Australia found that education modified the effect of lifestyle on DFLE.^66^ The current study showed a graded relationship between education and DLE (Figure 2). Both men and women with high WPA had lower education levels (Table 1). Again, the nonlinear relationship between WPA and DLE remained in women regardless of education level.

In addition to the potential selection bias mentioned above, one limitation of this study was that the results were based on microsimulation and transition probability rather than direct observation of life expectancy and transitions for each participant. Second, the physical activities were self‐reported, which may suffer from misclassification. However, self‐reported physical activity data are commonly used to study physical activity levels in large populations,^67^ and moderate correlations have been found between self‐reported and objectively measured physical activities.^68^ In addition, the MET assigned to each activity had been modified to reflect cultural differences according to locally collected data.^22^, ^69^ The cutoffs were determined by sex‐specific median or tertile (a relative measure) to reduce the impacts from absolute values. Third, physical activity was assessed at baseline and did not consider changes over the follow‐up period. A previous study suggested that consistently high physical activity levels over time were associated with lower risks of disability, worsening disability, hospitalization, and mortality, whereas low and decreased physical activity levels were associated with higher risks of adverse outcomes.^70^ Fourth, disability was assessed at baseline and reassessed 5–6 years later. The transition to disability may not reflect exactly when the event happened. Finally, we used ADL and IADL to define disability. Other studies have used only ADL,^71^ IADL disability,^62^ self‐rated health,^17^, ^18^ self‐reported medical conditions,^16^, ^18^ or medical conditions with disability weights to summarize DFLE and DLE. Although DFLE and DLE were meant to compare health statuses between populations, direct comparisons are difficult to make across studies that used different definitions. Nevertheless, our results were consistent with those studies that showed that LTPA was associated with longer DFLE regardless of definition. Our study further demonstrated a nonlinear association between WPA and DLE in women.

A major strength of this study was that it was based on a relatively large prospective cohort study with repeated measurements of ADL and IADL. Furthermore, use of the Stochastic Population Analysis for Complex Events program enabled adjusting for confounders in the model, which is a limitation in studies using traditional MSLT. Compared to the Sullivan method, which reflects the current health compositions adjusted for current mortality and morbidity, MSLT‐based methods provide an expected health structure according to current mortality and morbidity conditions.^72^

In summary, we found that LTPA was associated with health in older adults; while high levels of WPA did not provide additional benefits, particularly in women. Our data also suggest that high levels of WPA resulted from long hours of farming, including growing rice, fruits, and vegetables. This result suggest that prolonged duration of work might not be beneficial for health in middle‐aged or older adults. Public policy should advocate that older people watch the type, amount, and intensity of activities they perform,^54^ as these often may go ignored during WPA. However, the current study is inadequate to recommend employment policy at an older age because WPA in the current study does not correspond to employment. Another study on employment and/or occupation with full consideration of social support, education, and/or socioeconomic status is needed for policies on working beyond retirement age.

## Disclosure statement

The authors declare no conflict of interest.

## Funding information

This work was supported by the National Health Research Institutes in Taiwan (grant numbers PH‐111‐SP‐01, PH‐111‐PP‐19).

## Supporting information


**FIGURE S1.** Flow chart of the recruitment.
**FIGURE S2.** Sample included in the analyses.
**FIGURE S3.** Disabled life expectancies at 65 years old by household income and work‐related physical activity levels in men and women.

**TABLE S1.** Metabolic equivalent (MET) by levels of breathing efforts.
**TABLE S2.** Baseline characteristics for participants by follow‐up status at wave 2.
**TABLE S3.** Total, disability‐free, and disabled life expectancies by total physical activity (leisure‐time and work‐related combined).
**TABLE S4.** Energy expenditure (kcal/week) from work‐related physical activity in the National Health Interview Survey in Taiwan, 2009.

## Data Availability

The data that support the findings of this study are available on request from the corresponding author upon reasonable request. The data are not publicly available due to privacy or ethical restrictions.
