# Supplementary material for: Impact of physical activity on disability‐free and disabled life expectancies in middle‐aged and older adults: Data from the healthy aging longitudinal study in Taiwan
Source: Geriatr Gerontol Int. 2024 Jan 2;24(Suppl 1):229–39. doi: 10.1111/ggi.14796 (PMC11503563; doi:10.1111/ggi.14796)
Supplement: Supplementary file 1 — FIGURE S1. Flow chart of the recruitment. FIGURE S2. Sample included in the analyses. FIGURE S3. Disabled life expectancies at 65 years old by household income and work‐related physical activity levels in men and women. [file GGI-24-229-s002.pptx]

## Slide 1
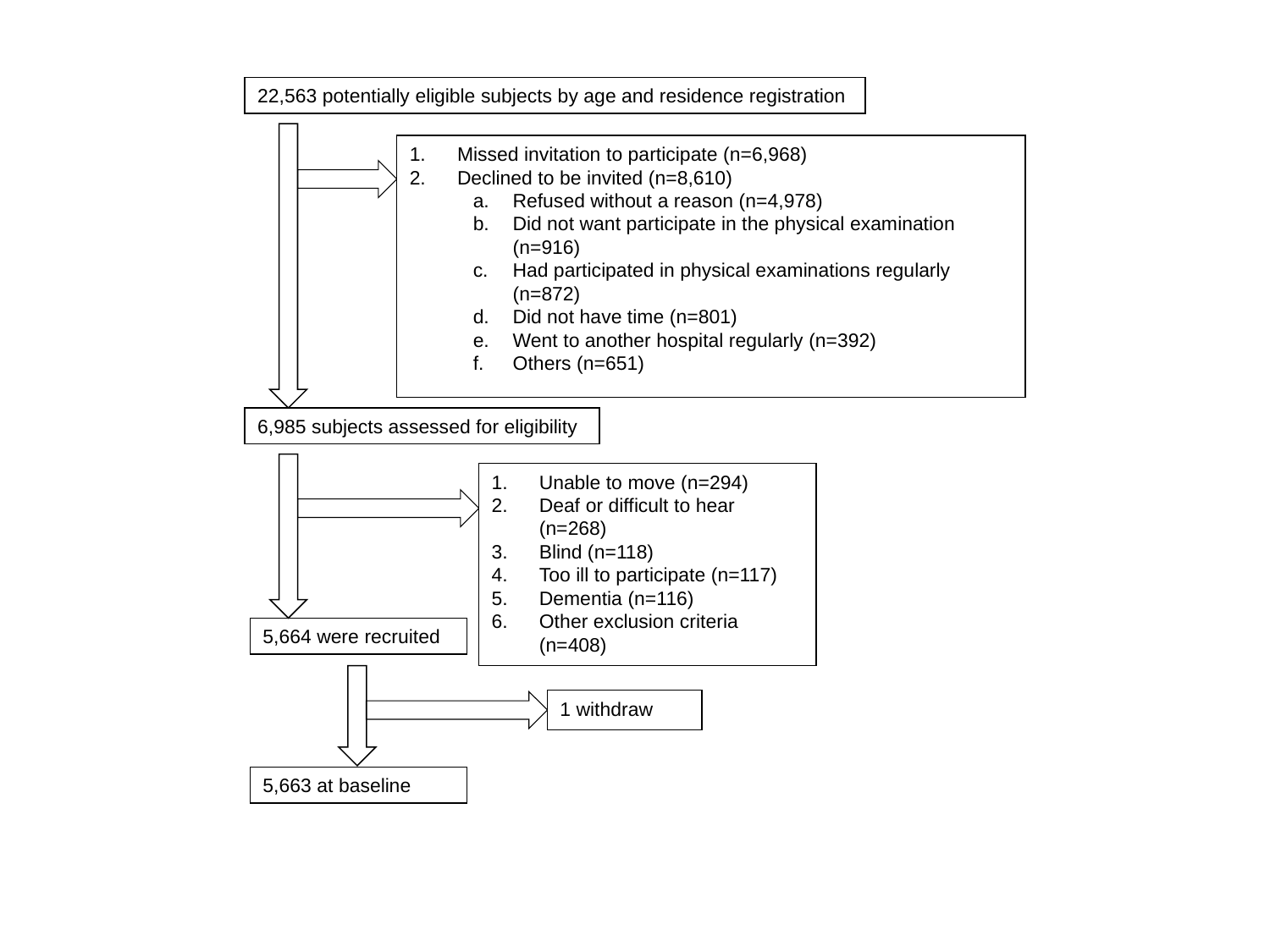

22,563 potentially eligible subjects by age and residence registration
Missed invitation to participate (n=6,968)
Declined to be invited (n=8,610)
Refused without a reason (n=4,978)
Did not want participate in the physical examination (n=916)
Had participated in physical examinations regularly (n=872)
Did not have time (n=801)
Went to another hospital regularly (n=392)
Others (n=651)
6,985 subjects assessed for eligibility
Unable to move (n=294)
Deaf or difficult to hear (n=268)
Blind (n=118)
Too ill to participate (n=117)
Dementia (n=116)
Other exclusion criteria (n=408)
5,664 were recruited
1 withdraw
5,663 at baseline

## Slide 2
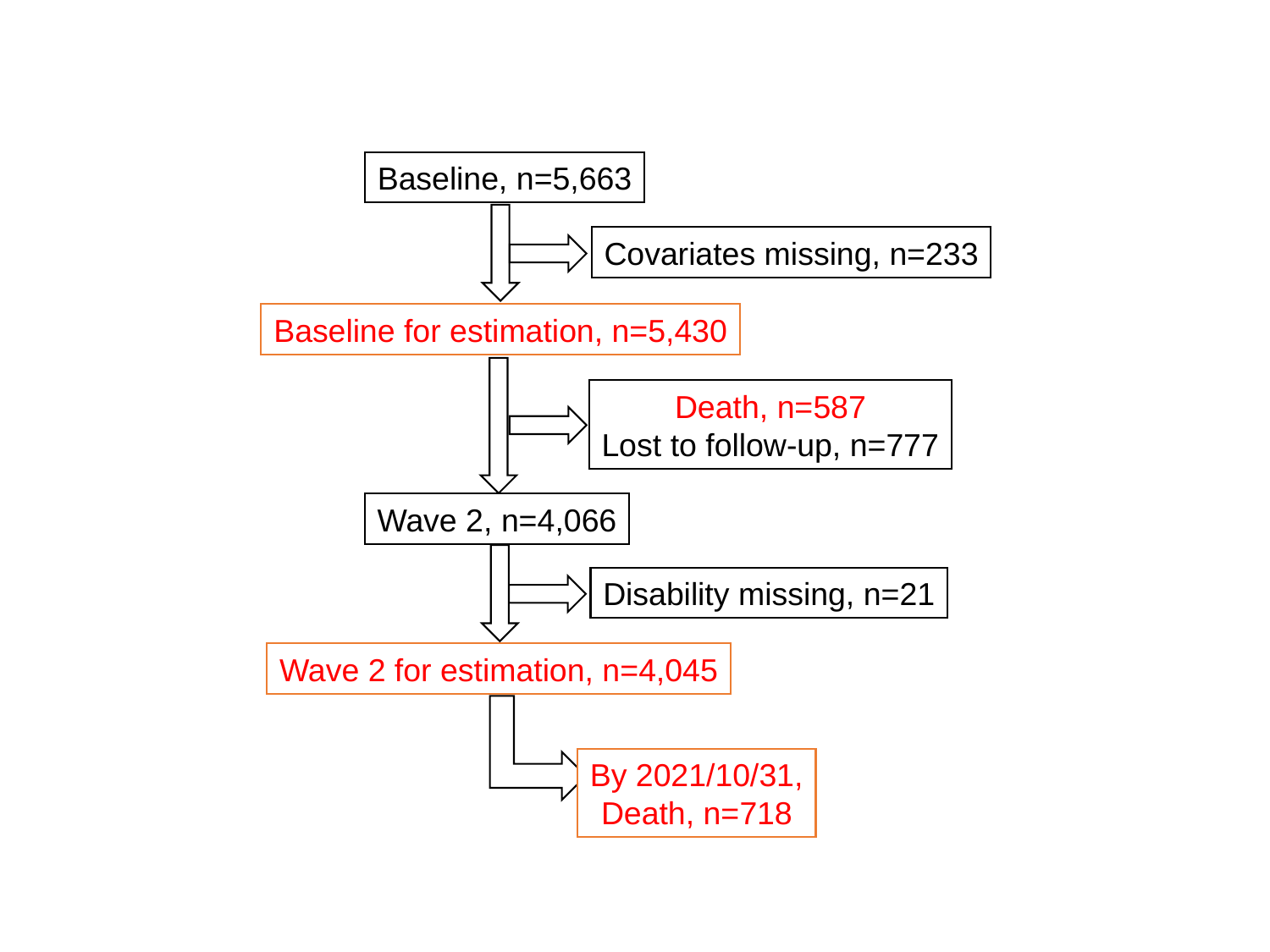

Baseline, n=5,663
Covariates missing, n=233
Baseline for estimation, n=5,430
Death, n=587
Lost to follow-up, n=777
Wave 2, n=4,066
Disability missing, n=21
Wave 2 for estimation, n=4,045
By 2021/10/31,
Death, n=718

## Slide 3
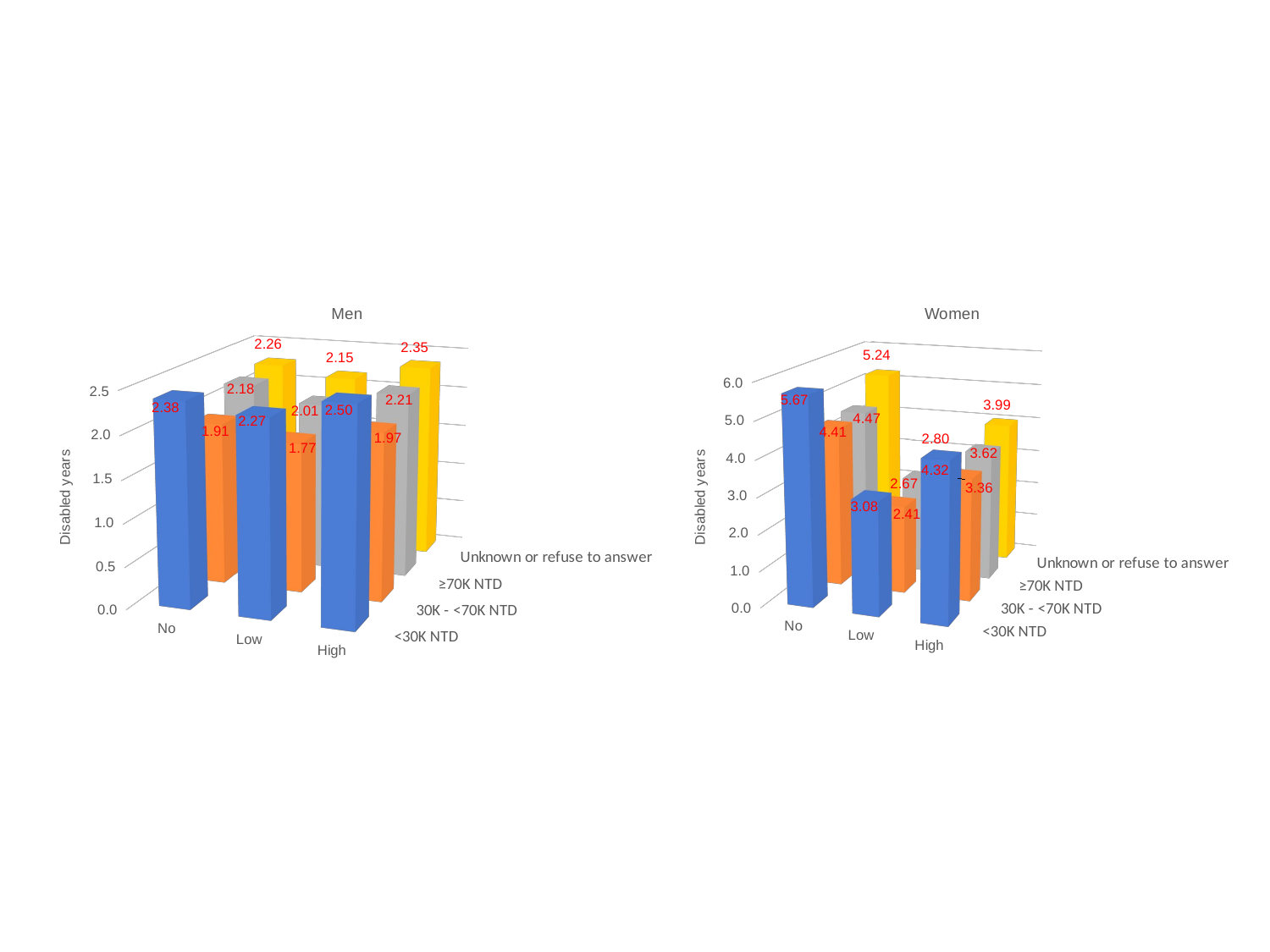

[unsupported chart]
[unsupported chart]
