# Supplementary material for: Impact of physical activity on disability‐free and disabled life expectancies in middle‐aged and older adults: Data from the healthy aging longitudinal study in Taiwan
Source: Geriatr Gerontol Int. 2024 Jan 2;24(Suppl 1):229–39. doi: 10.1111/ggi.14796 (PMC11503563; doi:10.1111/ggi.14796)
Supplement: Supplementary file 2 — TABLE S1. Metabolic equivalent (MET) by levels of breathing efforts. TABLE S2. Baseline characteristics for participants by follow‐up status at wave 2. TABLE S3. Total, disability‐free, and disabled life expectancies by total physical activity (leisure‐time and work‐related combined). TABLE S4. Energy expenditure (kcal/week) from work‐related physical activity in the National Health Interview Survey in Taiwan, 2009. [file GGI-24-229-s001.docx]

**Supplementary Table 1.** Metabolic equivalent (MET) by levels of breathing efforts

(A) Leisure time physical activities

| L1_1A_1 | Exercise | Without considering breathing efforts | 1 | 2 | 3 | 4 |
| --- | --- | --- | --- | --- | --- | --- |
|  |  |  | No change | Slight increase | Significant increase | Out of breath |
| 10 | Walking | 3.5 | 2.5 | 3.3 | 3.8 | 6.5 |
| 20 | Jogging | 6.0 | 3.3 | 3.8 | 5.8 | 7.4 |
| 30 | Brisk walking | 6.0 | 3.3 | 3.8 | 5.8 | 7.4 |
| 40 | Rope jumping | 10.0 | 8.8 | 8.8 | 11.8 | 12.3 |
| 50 | Swimming | 6.0 | 2.5 | 4.0 | 7.0 | 10.0 |
| 61 | Calisthenics | 4.0 | 2.8 | 3.8 | 6.0 | 8.0 |
| 62 | Ping Shuai Gong | 4.0 | 1.5 | 3.0 | 4.0 | 4.0 |
| 63 | Yoga | 4.0 | 2.0 | 2.5 | 3.3 | 4.0 |
| 71-77 | Chinese shadow boxing | 4.0 | 1.5 | 3.0 | 4.0 | 4.0 |
| 81 | Soccer | 7.0 | 2.5 | 4.0 | 8.0 | 10.0 |
| 82 | Golf | 4.5 | 3.0 | 4.3 | 4.8 | 5.3 |
| 83 | Badminton | 4.5 | 3.0 | 4.5 | 5.5 | 7.0 |
| 84 | Table tennis | 4.0 | 3.0 | 4.5 | 6.0 | 10.0 |
| 85 | Croquet | 4.0 | 3.0 | 4.5 | 5.5 | 7.5 |
| 86 | Tennis | 7.0 | 4.5 | 5.0 | 7.3 | 8.0 |
| 87 | Poll ball | 2.5 | 3.0 | 4.5 | 5.5 | 7.5 |
| 88 | Basketball | 6.0 | 4.5 | 6.0 | 8.0 | 9.3 |
| 89 | Other ball games | 5.5 | 3.0 | 4.5 | 5.5 | 7.5 |
| 90 | Aerobics | 6.0 | 2.5 | 5.0 | 7.0 | 8.5 |
| 91 | Chinese folk dance | 5.5 | 3.0 | 4.5 | 5.5 | 7.0 |
| 100 | Bicycling | 4.0 | 4.0 | 6.0 | 8.0 | 10.0 |
| 110 | Hiking | 8.0 | 4.0 | 6.3 | 7.3 | 8.3 |
| 120 | Weight training | 8.0 | 3.5 | 5.0 | 5.5 | 6.0 |
| 130 | Stair climbing | 8.0 | 4.0 | 5.0 | 6.5 | 8.0 |
| 140 | Hula hoop | 4.5 | 2.5 | 3.5 | 5.0 | 5.5 |
| 150 | Others | 3.0 | 3.0 | 4.5 | 6.0 | 8.0 |

(B) Work related physical activities

| L2_1A_1 | Work | Without considering breathing efforts | 1 | 2 | 3 | 4 |
| --- | --- | --- | --- | --- | --- | --- |
|  |  |  | No change | Slight increase | Significant increase | Out of breath |
| 1 | Farming | 2.5 | 2.0 | 3.8 | 4.8 | 7.8 |
| 2 | Heavy lifting | 8.0 | 8.0 | 8.0 | 8.0 | 8.0 |
| 3 | Fishing | 3.0 | 2.3 | 3.5 | 5.0 | 7.0 |
| 4 | Mining | 6.0 | 5.0 | 5.3 | 5.5 | 6.3 |
| 5 | Harvesting in the sea | 4.0 | 3.3 | 3.3 | 3.3 | 3.3 |
| 6 | Recycling | 4.0 | 4.0 | 4.0 | 4.0 | 4.0 |
| 7 | Construction work | 5.5 | 2.0 | 3.3 | 4.0 | 6.0 |
| 8 | Forestry worker | 8.0 | 4.5 | 5.0 | 8.0 | 17.5 |
| 9 | Hunting | 6.0 | 2.0 | 4.0 | 5.0 | 11.3 |
| 10 | Housekeeping | 3.5 | 2.3 | 3.3 | 3.8 | 4.5 |
| 11 | Walking to/off work | 4.0 | 2.0 | 3.5 | 3.5 | 4.3 |
| 12 | Bicycling to/off work | 4.0 | 3.5 | 4.0 | 5.8 | 6.8 |
| 13 | Baby sitting | 4.0 | 2.2 | 2.8 | 3.5 | 5.8 |
| 14 | Growing vegetables | 4.0 | 2.0 | 3.8 | 4.8 | 7.8 |
| 15 | Growing fruits | 4.0 | 2.0 | 3.8 | 4.8 | 7.8 |
| 16 | Others | 4.0 | 1.0 | 2.5 | 3.0 | 3.5 |

**Supplementary Table 2.** Baseline characteristics for participants by follow-up status at wave 2

|  | Completed (n=4066) | | Death before follow-up (n=587) | | Lost to follow-up (n=777) | |  |
| --- | --- | --- | --- | --- | --- | --- | --- |
|  | N | % | N | % | N | % | p |
| **Age (years, mean ± SD)** | 68.27 | 7.65 | 76.27 | 7.99 | 70.72 | 8.33 | <0.01 |
| **BMI (kg/m^2^, mean ± SD)^1^** | 24.60 | 3.43 | 23.89 | 3.69 | 24.74 | 3.65 | <0.01 |
| **Energy intake (kcal)** | 2053.39 | 743.55 | 1974.04 | 765.02 | 1948.81 | 752.20 | <0.01 |
| **Sex** |  |  |  |  |  |  |  |
| Men | 1901 | 46.8 | 374 | 63.7 | 308 | 39.6 | <0.01 |
| Women | 2165 | 53.2 | 213 | 36.3 | 469 | 60.4 |  |
| **Education levels** |  |  |  |  |  |  |  |
| Illiteracy | 337 | 8.3 | 88 | 15.0 | 123 | 15.8 | <0.01 |
| Primary school | 1750 | 43.0 | 283 | 48.2 | 384 | 49.4 |  |
| More than primary school | 1979 | 48.7 | 216 | 36.8 | 270 | 34.7 |  |
| **Household income** |  |  |  |  |  |  |  |
| <30K NTD | 1215 | 29.9 | 227 | 38.7 | 288 | 37.1 | <0.01 |
| 30K-<70K NTD | 546 | 13.4 | 71 | 12.1 | 79 | 10.2 |  |
| ≥70K NTD | 452 | 11.1 | 21 | 3.6 | 44 | 5.7 |  |
| Unknown and refused to answer | 1853 | 45.6 | 268 | 45.7 | 366 | 47.1 |  |
| **Smoking** |  |  |  |  |  |  |  |
| Never | 2999 | 73.8 | 317 | 54.0 | 571 | 73.5 | <0.01 |
| Former | 608 | 15.0 | 148 | 25.2 | 103 | 13.3 |  |
| Current | 459 | 11.3 | 122 | 20.8 | 103 | 13.3 |  |
| **Drinking** |  |  |  |  |  |  |  |
| Never | 2408 | 59.2 | 335 | 57.1 | 540 | 69.5 | <0.01 |
| Former | 388 | 9.5 | 125 | 21.3 | 56 | 7.2 |  |
| Current | 1270 | 31.2 | 127 | 21.6 | 181 | 23.3 |  |
| **Physical activity at leisure time** |  |  |  |  |  |  |  |
| Low | 1243 | 30.6 | 238 | 40.5 | 288 | 37.1 | <0.01 |
| Mediate | 1363 | 33.5 | 214 | 36.5 | 259 | 33.3 |  |
| High | 1460 | 35.9 | 135 | 23.0 | 230 | 29.6 |  |
| **Physical activity at work** |  |  |  |  |  |  |  |
| No | 2996 | 73.8 | 544 | 79.4 | 526 | 76.6 | <0.01 |
| Low | 479 | 11.8 | 52 | 7.6 | 56 | 8.2 |  |
| High | 583 | 14.4 | 89 | 13.0 | 105 | 15.3 |  |
| **Social network** |  |  |  |  |  |  |  |
| ≥8 | 2095 | 51.5 | 205 | 34.9 | 336 | 43.2 | <0.01 |
| 6-7 | 1133 | 27.9 | 165 | 28.1 | 211 | 27.2 |  |
| 0-5 | 838 | 20.6 | 217 | 37.0 | 230 | 29.6 |  |
| **CESD** |  |  |  |  |  |  |  |
| <16 | 3871 | 95.2 | 532 | 90.6 | 722 | 92.9 | <0.01 |
| ≥16 | 195 | 4.8 | 55 | 9.4 | 55 | 7.1 |  |
| **Prevalence of chronic conditions** |  |  |  |  |  |  |  |
| Disability | 492 | 12.1 | 199 | 33.9 | 166 | 21.4 | <0.01 |
| Hypertensive medication | 1593 | 39.2 | 302 | 51.4 | 352 | 45.3 | <0.01 |
| Dyslipidemia medication | 583 | 14.3 | 96 | 16.4 | 117 | 15.1 | 0.41 |
| Diabetic medication | 614 | 15.1 | 149 | 25.4 | 144 | 18.5 | <0.01 |
| Metabolic syndrome | 1917 | 47.5 | 305 | 53.2 | 393 | 53.7 | <0.01 |
| Stroke | 173 | 4.3 | 67 | 11.4 | 46 | 5.9 | <0.01 |
| Cancer | 218 | 5.4 | 58 | 9.9 | 42 | 5.4 | <0.01 |
| Gout | 431 | 10.6 | 84 | 14.3 | 86 | 11.1 | 0.03 |
| Hip fracture | 52 | 1.3 | 21 | 3.6 | 17 | 2.2 | <0.01 |
| Other musculoskeletal diseases | 1727 | 42.5 | 223 | 38.0 | 346 | 44.5 | 0.05 |

**Supplementary Table 3.** Total, disability-free, and disabled life expectancies by total physical activity (leisure-time and work-related combined)

|  |  |  | | |  | | |  | | | Net gain or loss | | | | | | | | |
| --- | --- | --- | --- | --- | --- | --- | --- | --- | --- | --- | --- | --- | --- | --- | --- | --- | --- | --- | --- |
|  |  | Total life expectancy | | | Disability-free life expectancy | | | Disabled life expectancy | | | Total life expectancy | | | Disability-free life expectancy | | | Disabled life expectancy | | |
|  |  | Years | 95% CI | | Years | 95% CI | | Years | 95% CI | | Years | 95% CI | | Years | 95% CI | | Years | 95% CI | |
|  | **Men** |  |  |  |  |  |  |  |  |  |  |  |  |  |  |  |  |  |  |
| At 55 | Low | 23.92 | (22.88, | 24.95) | 21.27 | (19.99, | 22.54) | 2.65 | (1.87, | 3.43) | Ref |  |  | Ref |  |  | Ref |  |  |
|  | Moderate | 25.90 | (24.73, | 27.08) | 23.76 | (22.48, | 25.05) | 2.14 | (1.36, | 2.93) | 1.99 | (0.42, | 3.55) | 2.49 | (0.68, | 4.31) | -0.51 | (-1.62, | 0.60) |
|  | High | 26.19 | (25.14, | 27.24) | 23.93 | (22.82, | 25.03) | 2.27 | (1.69, | 2.85) | 2.27 | (0.80, | 3.75) | 2.66 | (0.97, | 4.35) | -0.38 | (-1.36, | 0.59) |
| At 65 | Low | 15.74 | (14.97, | 16.51) | 13.21 | (12.34, | 14.08) | 2.53 | (1.94, | 3.12) | Ref |  |  | Ref |  |  | Ref |  |  |
|  | Moderate | 17.38 | (16.45, | 18.31) | 15.35 | (14.35, | 16.34) | 2.04 | (1.41, | 2.66) | 1.64 | (0.44, | 2.85) | 2.14 | (0.82, | 3.45) | -0.49 | (-1.35, | 0.37) |
|  | High | 17.67 | (16.79, | 18.55) | 15.52 | (14.65, | 16.39) | 2.15 | (1.65, | 2.65) | 1.93 | (0.76, | 3.10) | 2.31 | (1.08, | 3.54) | -0.38 | (-1.15, | 0.39) |
| At 75 | Low | 9.11 | (8.61, | 9.61) | 6.71 | (6.17, | 7.24) | 2.41 | (1.97, | 2.84) | Ref |  |  | Ref |  |  | Ref |  |  |
|  | Moderate | 10.34 | (9.65, | 11.03) | 8.33 | (7.63, | 9.04) | 2.00 | (1.53, | 2.48) | 1.22 | (0.37, | 2.08) | 1.63 | (0.74, | 2.51) | -0.40 | (-1.05, | 0.24) |
|  | High | 10.52 | (9.81, | 11.23) | 8.51 | (7.86, | 9.16) | 2.01 | (1.57, | 2.46) | 1.41 | (0.54, | 2.28) | 1.80 | (0.96, | 2.65) | -0.39 | (-1.02, | 0.23) |
|  | **Women** | | | |  |  |  |  |  |  |  |  |  |  |  |  |  |  |  |
| At 55 | Low | 29.69 | (28.63, | 30.76) | 23.55 | (22.08, | 25.01) | 6.15 | (5.08, | 7.22) | Ref |  |  | Ref |  |  | Ref |  |  |
|  | Moderate | 30.15 | (29.06, | 31.24) | 25.63 | (24.55, | 26.70) | 4.52 | (3.65, | 5.38) | 0.45 | (-1.07, | 1.98) | 2.08 | (0.27, | 3.90) | -1.63 | (-3.01, | -0.25) |
|  | High | 29.75 | (28.60, | 30.89) | 25.03 | (23.81, | 26.25) | 4.72 | (3.55, | 5.88) | 0.05 | (-1.51, | 1.62) | 1.48 | (-0.42, | 3.39) | -1.43 | (-3.01, | 0.15) |
| At 65 | Low | 20.14 | (19.19, | 21.08) | 14.43 | (13.29, | 15.58) | 5.70 | (4.84, | 6.57) | Ref |  |  | Ref |  |  | Ref |  |  |
|  | Moderate | 20.63 | (19.64, | 21.62) | 16.37 | (15.45, | 17.28) | 4.26 | (3.56, | 4.96) | 0.50 | (-0.87, | 1.86) | 1.93 | (0.47, | 3.40) | -1.44 | (-2.55, | -0.33) |
|  | High | 20.28 | (19.21, | 21.36) | 15.84 | (14.80, | 16.88) | 4.44 | (3.49, | 5.39) | 0.15 | (-1.28, | 1.57) | 1.41 | (-0.14, | 2.96) | -1.26 | (-2.54, | 0.03) |
| At 75 | Low | 11.71 | (10.98, | 12.43) | 7.04 | (6.31, | 7.76) | 4.67 | (4.02, | 5.32) | Ref |  |  | Ref |  |  | Ref |  |  |
|  | Moderate | 11.97 | (11.17, | 12.78) | 8.38 | (7.70, | 9.07) | 3.59 | (3.05, | 4.13) | 0.27 | (-0.82, | 1.35) | 1.35 | (0.35, | 2.35) | -1.08 | (-1.92, | -0.24) |
|  | High | 11.87 | (10.92, | 12.82) | 8.03 | (7.26, | 8.80) | 3.84 | (3.09, | 4.60) | 0.17 | (-1.03, | 1.36) | 1.00 | (-0.06, | 2.05) | -0.83 | (-1.82, | 0.17) |

**Supplementary Table 4.** Energy expenditure (kcal/week) from work related physical activity in the National Health Interview Survey in Taiwan, 2009

|  | Men | | | | | Women | | | | |
| --- | --- | --- | --- | --- | --- | --- | --- | --- | --- | --- |
| Age | p5 | p25 | median | p75 | p95 | p5 | p25 | median | p75 | p95 |
| 15-24 | 46 | 302 | 1,091 | 5,214 | 20,480 | 21 | 77 | 206 | 800 | 6,624 |
| 25-34 | 102 | 630 | 2,784 | 8,580 | 29,792 | 40 | 133 | 393 | 2,180 | 11,424 |
| 35-44 | 115 | 710 | 3,024 | 7,773 | 21,840 | 36 | 165 | 555 | 2,807 | 17,280 |
| 45-54 | 115 | 656 | 3,000 | 7,638 | 23,184 | 43 | 204 | 724 | 4,088 | 15,962 |
| 55-64 | 92 | 900 | 2,360 | 6,560 | 17,472 | 49 | 294 | 1,248 | 3,960 | 19,488 |
| 65-74 | 125 | 789 | 2,115 | 4,549 | 13,440 | 50 | 218 | 966 | 2,785 | 7,728 |
| 75+ | 100 | 483 | 1,381 | 2,827 | 11,172 | 20 | 178 | 600 | 1,775 | 7,056 |
